# Association of single nucleotide polymorphisms in *FILIP1-SENP6* and *FTO* with temporomandibular joint osteoarthritis: clinical and in silico study

**DOI:** 10.1038/s41598-026-47515-5

**Published:** 2026-04-10

**Authors:** Emi Ono, Ryota Takaoka, Kotaro Kuyama, Masayuki Ono, You Ueda, Shion Morika, Rie Yamamoto, Hirofumi Yatani, Shoichi Ishigaki, Takafumi Kato, Yuka Uchiyama, Hiroaki Shimamoto, Masahiro Nishimura

**Affiliations:** 1 Department of Regenerative Prosthodontics, Graduate School of Dentistry, The University of Osaka, 1-8 Yamadaoka, Suita, 565-0871 Osaka Japan; 2 Department of Microbiology, Graduate School of Dentistry, The University of Osaka, 1-8 Yamadaoka, Suita, 565-0871 Osaka Japan; 3 Bioinformatics Research Unit, Graduate School of Dentistry, The University of Osaka, 1-8 Yamadaoka, Suita, 565-0871 Osaka Japan; 4 Department of Oral Physiology, Graduate School of Dentistry, The University of Osaka, 1-8 Yamadaoka, Suita, 565-0871 Osaka Japan; 5 Department of Oral and Maxillofacial Radiology, Graduate School of Dentistry, The University of Osaka, 1-8 Yamadaoka, Suita, 565-0871 Osaka Japan; 6 Center of Excellence in Precision Medicine and Digital Health, Department of Physiology, Faculty of Dentistry, Chulalongkorn University, Bangkok, 10330 Thailand

**Keywords:** Temporomandibular joint osteoarthritis, Single nucleotide polymorphisms, *FILIP1*, *SENP6*, *FTO*, In silico analysis, Biomarkers, Computational biology and bioinformatics, Diseases, Genetics, Rheumatology

## Abstract

This study investigated the association between temporomandibular joint osteoarthritis and specific single-nucleotide polymorphisms. We also explored the potential functional impacts of these polymorphisms using in silico methods. 354 patients with temporomandibular disorders were classified into osteoarthritis and without osteoarthritis according to the presence of degenerative bone changes in the magnetic resonance imaging including osteophytes, erosion, subchondral cysts, and atrophy. Genomic DNA was extracted and genotyping was performed to determine 16 alleles. Multivariate analysis was performed to explore the polymorphisms associated with temporomandibular joint osteoarthritis, followed by in silico analysis to predict the functional relevance of the polymorphisms. Multivariate analysis identified that rs9350591 (between *FILIP1* and *SENP6*) and rs8044769 (*FTO*) are potentially associated with temporomandibular joint osteoarthritis. In silico analyses revealed that rs9350591 affects transcription factor binding, whereas rs8044769 alters the predicted score as a splicing site. These findings suggest that genetic factors, particularly rs9350591 and rs8044769, are associated with temporomandibular joint osteoarthritis among patients with temporomandibular disorders. This underscores the importance of genetic studies for understanding temporomandibular joint osteoarthritis. This study provides the potential genetic clues for temporomandibular joint osteoarthritis and insights into the genetic and molecular mechanisms underlying this disease.

**Supplementary Information:**

The online version contains supplementary material available at 10.1038/s41598-026-47515-5.

## Background

Osteoarthritis (OA) is a complex degenerative joint disease characterized by structural alterations in the articular cartilage. OA has a significant impact on patients’ health span, life expectancy, and quality of life due to pain^1^. OA is the most prevalent bone and joint disease, affecting 595 million individuals in 2020, representing 7.6% of the global population. The total number of patients affected in 2023 will increase by 132.2% compared to that in 1990^2^. OA can develop in all joints, and the temporomandibular joint (TMJ) is no exception.

OA that develops in the TMJ is referred to as the temporomandibular joint osteoarthritis (TMJOA). The pathological process of TMJOA is characterized by the destruction of cartilage and bone structure in the mandibular condyle, mandibular fossa, and articular eminence. It is considered the final stage of a temporomandibular disorder (TMD)^3,4^. Some studies have reported that TMJOA is found in 17.4% of adults and older individuals and 0.4% of children^5^. Joint noise (mainly crepitus), pain, and dysfunction are general symptoms of TMJOA^6,7^. In severe cases, these symptoms significantly reduce a patient’s quality of life^8^. In addition, refractory cases of TMJOA do not achieve the desired outcome even after appropriate treatment^9^. Factors contributing to the development of knee OA and TMJOA include hormonal balance (particularly estrogen levels), inflammation, mechanical stress, and genetic predisposition. For example, in knee OA, regulation mediated by estrogen-related receptors (ERRs) has been reported, with suppression of ERRα or overexpression of ERRγ suggested to contribute to cartilage destruction and inflammation. Additionally, ERRs are considered to be involved in TMJOA, although their distribution differs from that in the knee joint^10^.

In a normal TMJ, the thinnest intermediate portion of the disc is located between the condyle and fossa, facilitating smooth jaw movement and protecting the cartilage on the bone surfaces. However, temporomandibular disc is often displaced anteriorly^11^. In the initial stages of intraarticular disorders, the displaced articular disc returns to its normal position with mouth opening (disc displacement with reduction, DDwR). In the later stages, disc displacement remained throughout the mouth opening (disc displacement without reduction: DDwoR). DDwoR is a major risk factor for degenerative bone changes in the mandibular condyle^12,13^. It has also been reported that weakening of the collateral ligament due to continuous stress can lead to DDwoR^14,15^. However, not all patients with DDwoR develop TMJOA, and the onset of TMJOA remains unpredictable.

Genome-wide association studies have suggested that several single-nucleotide polymorphisms (SNPs) may be risk factors for limb OA, and SNPs are currently used as biomarkers for various human diseases. For example, *BRCA1* and *BRCA2* gene polymorphisms are used to identify individuals at substantial risk of breast and ovarian cancers, and preventive surgeries or regular monitoring are implemented for high-risk patients^16^. Additionally, in lung cancer treatment, specific mutations or SNPs in *EGFR* are used to predict the sensitivity to tyrosine kinase inhibitors^17^.

With recent advancements in next-generation sequencing technologies, it has become possible to analyze genetic mutations in non-coding regions, emphasizing the importance of evaluating their impact on disease development. In a study by Jaganathan et al. (2019), a deep learning model called “SpliceAI” was developed, demonstrating high accuracy in predicting splicing abnormalities from genomic sequences^18^. Validation using RNA-Seq data experimentally confirmed over 75% of the predictions, proving that these mutations play critical roles in disease pathogenesis. This highlights that in silico analysis is a powerful tool for efficiently screening disease-related mutations and narrowing targets for experimental validation. Additionally, in silico analysis using the ESEfinder tool identified mutations in exonic splicing enhancers (ESEs), which were subsequently validated through in vitro assays and experiments with patient-derived samples, confirming a causal relationship with aberrant splicing^19^. These findings demonstrated that ESEfinder is a valuable tool for predicting splicing abnormalities and identifying disease-related mutations.

Some studies have identified SNPs associated with OA and anterior cruciate ligament injury, which are known risk factors for knee OA^20–25^. Genetic analysis is expected to expand the possibilities of disease prediction and tailor-made medicine based on individual genetic predispositions. However, only a limited number of investigations of TMJOA have focused on genetic predispositions, including SNPs.

Based on this background, we hypothesized that genetic factors are associated with TMJOA and disc abnormalities. This study aimed to investigate the association between TMJOA and SNPs previously shown to contribute to limb OA and OA-related diseases using magnetic resonance (MR) imaging for OA diagnosis and disc abnormalities. In addition, we attempted to predict the potential function of the SNP alleles in pathogenesis by utilizing in silico-based analyses, which have been applied in multiple studies and have been demonstrated to assist the understanding of the mechanisms of diseases^18,19^. This study may provide new insights into pathogenic mechanisms, therapeutic targets, and disease prediction in TMJOA.

## Methods

### Subjects

A total of 301 patients who visited the Osaka University Dental Hospital, with a chief complaint of TMJ symptoms (at least one of restricted mouth opening, joint noise, or pain) between January 2019 and November 2022 were selected for this study. Five patients were excluded from the study population of 301 patients after they were diagnosed with rheumatoid arthritis, synovial chondromatosis, masticatory muscle tendon-aponeurosis hyperplasia, or Ehlers-Danlos syndrome. Next, from the database of 678 patients who presented to the hospital between 2015 and 2018 and underwent TMJ MR imaging, we selected 38 patients with severe degenerative joint disease, including osteophytes, erosion, atrophy, and subchondral cysts in the mandibular condyle, and 42 patients with normal TMJs without any disc displacements and degenerative bone changes. A letter was sent to each patient requesting them to participate in the study. Consent to participate in the study was obtained from 31 of 38 patients with severe degenerative joint disease of the mandibular condyle and 27 of 42 patients with normal TMJs.

**Fig. 1:**
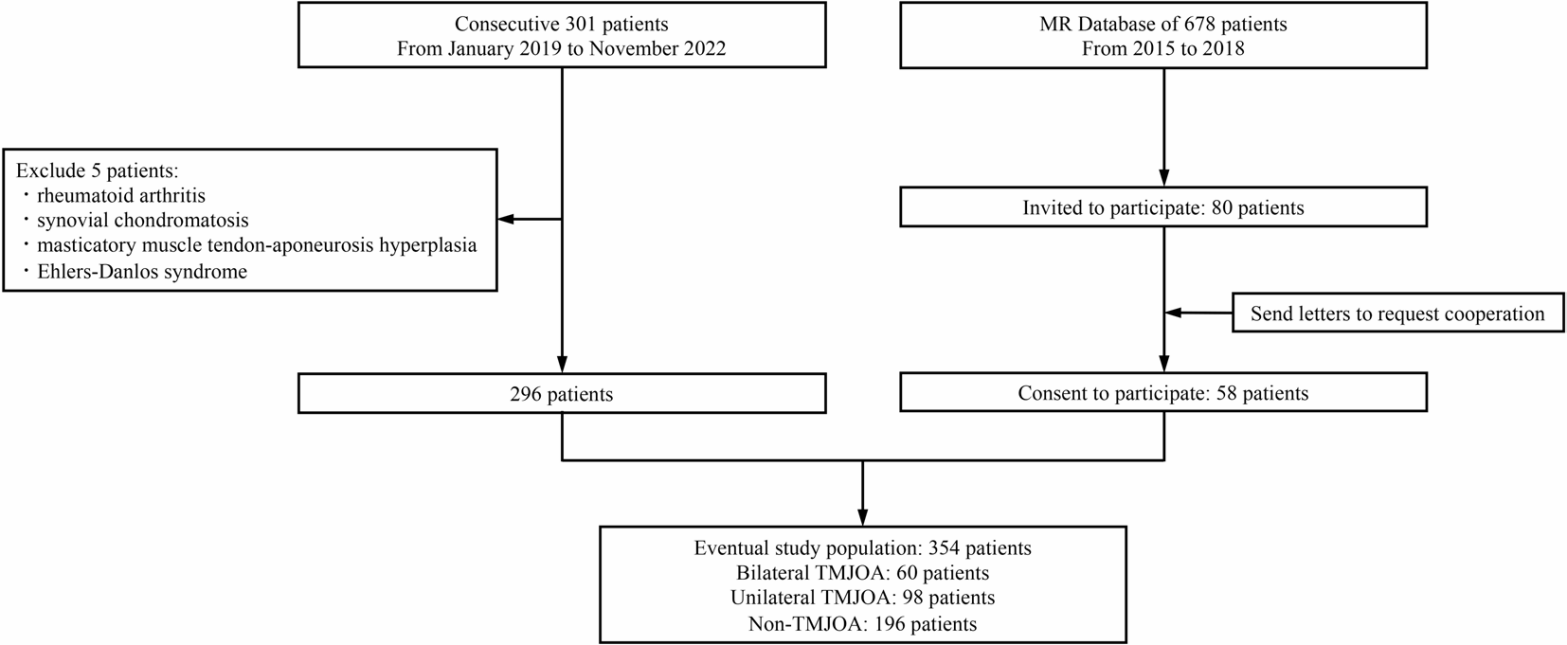
Flowchart for the selection of the study participants. TMJOA, temporomandibular joint osteoarthritis.

The final study population consisted of 60 patients with TMJOA on both sides, 98 patients with unilateral TMJOA, and 196 patients without TMJOA (Fig. 1). The study procedures were approved by the Ethical Review Board of Osaka University (H30-E11). Informed consent for study participation was obtained from all subjects involved in this study. This study complied with the STROBE statement, and all procedures were performed in accordance with the 1964 Helsinki Declaration and its later amendments.

### Magnetic resonance imaging

A Signa HDxt 1.5T MRI system (GE Healthcare, Chicago, IL, USA) was used for imaging at the Department of Dental Radiology. MR imaging was performed on all the subjects as previously described^26^. A fast spin echo sequence was used to image the sagittal and coronal planes of the TMJs. The sagittal plane was oriented perpendicularly to the long axis of the mandibular head. Proton-weighted images of the bilateral sagittal and coronal planes during mouth closure were acquired using the following parameters: TR = 2,500 ms, TE = 20 ms, NEX = 2, and ETL = 8. Fat-suppressed T2-weighted images in the bilateral sagittal and coronal planes during mouth closure were acquired using the following sequence: TR = 2,000 ms, TE = 85 ms, NEX = 3, and ETL = 16. Proton-weighted images (TR = 800 ms, TE = 24 ms, NEX = 2, and ETL = 4) were also acquired with the mouth open and closed to assess the articular disc reduction. The remaining parameters were as follows: field of view (FOV) = 10 cm, matrix size = 256 × 160 or 256 × 128, and slice thickness = 3 mm. Blocks of 5-mm thickness increments were prepared, and the thickest block from when the patient had an open mouth was selected for MR examination.

### Diagnosis of disc displacement and TMJOA

Two oral and maxillofacial radiologists who were blinded to clinical and genetic information independently conducted MR assessments. In case of a disagreement between their diagnoses, they discussed and the final decision was made by consensus. All slices in the sagittal and coronal planes of the MR scans were evaluated to diagnose the condition of the articular disc and degenerative bone changes in the mandibular condyle. The classification of positional disc abnormalities, dynamic disc abnormalities, and degenerative bone changes of the condyles is as follows^26^:

1. Classification of positional disc abnormalities.
  1. Normal: the condylar head is located in the narrowest portion of the articular disc in the sagittal plane when the mouth is closed and the articular disc exhibits a biconcave morphology.
  2. Disc displacement.

Disc displacement is defined as any of the following three states.

i. Sideway displacement: dislocation to the lateral side beyond the medial pole or lateral pole in the coronal plane and at least one or more planes, including the condylar head in the sagittal plane, where it is confirmed that the disc has disappeared.
ii. Anterior displacement: at least one sagittal plane in which the posterior thickened portion of the disc is located anterior to the 11:30 direction of the condylar head is confirmed.
iii. Posterior displacement: in the sagittal plane, the thickened anterior portion of the disc is positioned posterior to the condylar head.

(2) Classification of dynamic disc abnormalities.
  1. Normal: the mandibular condyle is positioned in the narrow central part of the articular disc on the sagittal plane in both the open- and closed-mouth positions.
  2. DDwR: the articular disc is displaced in the closed-mouth position; however, in the open-mouth position, the relationship between the mandibular condyle, mandibular fossa, and the articular disc is normal.
  3. DDwoR: the articular disc is displaced in the closed-mouth position and remains displaced in the open-mouth position.
(3) Degenerative bone changes in the condylar head.

The study samples were assigned to the joints with OA if at least one of the following four degenerative bone changes was found on MR examinations: osteophytes, erosion, subchondral cysts, and atrophy. Those without any of these features were assigned to the joints without OA. Flattening and concavity are not included in the diagnostic criteria for TMJOA^27,28^.

### Genomic DNA extraction and SNP genotyping

DNA samples were collected non-invasively from the buccal mucosa of the subjects using FLOQSwabs^®^ (Copan, Brescia, Italy). The samples were then stored frozen at −20 ˚C. SNP genotyping using MassARRAY^®^ was performed at Takara Bio (Shiga, Japan) to determine the following alleles: rs3815148 in *COG5*, rs11177 in *GNL3*, rs4836732 in *ASTN2*, rs9350591 in *FILIP1/SENP6*, rs10492367 in *KLHDC5/PTHLH*, rs835487 in *CHST11*, rs12107036 in *TP63*, rs8044769 in *FTO*, rs10948172 in *SUPT3H*, rs12982744 in *DOT1L*, rs6094710 in *NCOA3*, rs11718863 in *DVWA*, rs4730250 in *DUS4L*, rs516115 in *DCN*, rs2862851 in *TGFA*, and rs143383 in *GDF5*.

Genotyping was performed blindly by laboratory personnel at Takara Bio, and its accuracy was validated in advance using samples from another 22 cases, showing a degree of agreement of 100%. Genotyping quality control was performed by evaluating genotype call rates for each SNP and sample. All SNPs showed high genotype call rates (> 97%), and samples with a ‘no call’ for any of the analyzed SNPs were excluded from the final dataset to ensure the highest data integrity. Hardy–Weinberg equilibrium was assessed in the study population, and no SNP showed substantial deviation (*P* > 0.05).

### Data and statistical analyses

Statistical analyses were conducted using R version 4.3.1 (2023-06-16; R Project for Statistical Computing, Vienna, Austria). The concordance between diagnoses in the MR images made by the two examiners was evaluated using the kappa coefficient. The chi-square test was used to assess differences between patients with and without TMJOA in terms of sex, positional disc abnormalities, dynamic disc abnormalities, and allele distributions of the analyzed SNPs. For positional disc abnormalities, dynamic disc abnormalities, and TMJOA, when discordant phenotypes were observed between the two joints of an individual, the more severe phenotype was used for the analysis. The difference in age distribution between the groups was assessed using the Mann–Whitney U test.

A binomial logistic regression model assuming individual-level random effects was constructed using Bayesian modeling with the R package brms^29^. Specifically, the presence or absence of TMJOA was set as the outcome variable, whereas age, sex, dynamic disc abnormality (a three-level ordinal variable), and genomic alleles (three-level ordinal variables ordered as homozygous, heterozygous, and other homozygous genotypes) were used as explanatory variables. Since a pair of positional disc abnormalities and dynamic disc abnormalities exhibited a high coefficient (> 0.7), the positional disc abnormalities were excluded from the model. The model included an intercept for individual random effects. The prior distributions followed default settings: a uniform distribution for the explanatory variables and for the intercept and variance, a t-distribution with three degrees of freedom, a mean of zero, and a variance of 2.5. Calculations were performed using chains = 2 and 5000 iterations. In this model, the linear component represents the primary allele dosage effect across genotypes, whereas the quadratic component captures non-linear deviations from additivity. Therefore, the linear component was used as the primary criterion for identifying associated SNPs. Coefficients of the linear component with posterior probability above 0.95 were assumed to potentially contribute to the pathology. Because all participants had TMD, the detected associations with TMJOA should be interpreted as factors associated with the development of osseous degeneration within a symptomatic TMD population. Additionally, to improve interpretability, we performed sensitivity analyses under alternative inheritance codings, including an additive model and dominant/recessive models. Case-control statistical power estimation was performed using R package genpwr v.1.0.4.

### In silico analysis

Sets of TMJOA-associated SNPs with linkage disequilibrium were determined using the jMorp database^30^. We determined variants with linkage disequilibrium coefficients (r^2^) more than 0.8 as co-occurring variations. The function of the intergenic variations was predicted using RegulomeDB, SNP2TFBS, and Transcription Factor Affinity Prediction (TRAP) Web Tools^31–33^. In the TRAP analysis, the difference between two sequences (sTRAP) was employed, and we compared the transcription factor (TF) binding affinity in TF matrices in which *p-*values in both the wild-type and mutant sequences were below 0.05. In silico analyses of the intronic variants were performed using SpliceAI-lookup and ESEfinder v.3.0^34–36^. ESEfinder calculates the exonic splicing enhancer (ESE) motif scores for five serine/arginine-rich proteins, 5’ and 3’ splice sites, and branch sites.

## Results

Table 1 presents the descriptive statistics of this study. The kappa coefficients for examiner concordance in the diagnosis of TMJOA, positional disc abnormalities, and dynamic disc abnormalities were 0.89, 0.92, and 0.82, respectively (*P* < 0.05). The chi-square and Mann–Whitney U tests showed statistically significant differences in sex, age, positional disc abnormalities, dynamic disc abnormalities, and rs12107036 in *TP63* between the patients with and without TMJOA.

**Table 1:** Study population characteristics.

	Patients without OA (*n* = 196)	Patients with OA (*n* = 158)	*P*-value
Sex: *n* [%]	Male 60 [30.6%] Female 136 [69.4%]	Male 28 [17.7%] Female 130 [82.3%]	0.008
Age (y): median [IQR]	47 [26.75–62]	54 [42–64.75]	0.002
Positional disc abnormalities: n	Absent: 45 Present: 151	Absent: 2 Present: 156	< 0.001
Dynamic disc abnormalities: n	Normal: 43 DDwR: 92 DDwoR: 61	Normal: 2 DDwR: 12 DDwoR: 144	< 0.001
rs3815148 (*COG5*) AA/AC/CC: n	158/37/1	131/25/2	0.574
rs11177 (*GNL3*) CC/CT/TT: n	55/96/45	39/76/43	0.598
rs4836732 (*ASTN2*) TT/TC/CC: n	40/93/63	22/90/46	0.143
rs9350591 (*FILIP1/SENP6*) CC/CT/TT: n	135/60/1	118/37/3	0.169
rs835487 (*CHST11*) AA/AG/GG: n	87/87/22	73/69/16	0.918
rs12107036 (*TP63*) AA/AG/GG: n	66/106/24	59/60/39	0.002
rs8044769 (*FTO*) CC/CT/TT: n	92/84/20	86/53/19	0.202
rs10948172 (*SUPT3H*) AA/AG/GG: n	188/8/0	145/13/0	0.157
rs12982744 (*DOT1L*) CC/CG/GG: n	106/71/19	78/68/12	0.397
rs11718863 (*DVWA*) AA/AT/TT: n	62/96/38	45/81/32	0.814
rs516115 (*DCN*) AA/AG/GG: n	91/76/29	72/68/18	0.558
rs2862851 (*TGFA*) TT/TC/CC: n	72/97/27	72/68/18	0.271
rs143383 (*GDF5*) AA/AG/GG: n	123/66/7	93/55/10	0.441

Statistical differences between the patients with without TMJOA were examined using the Mann–Whitney and chi-square tests for age and other variables, respectively. Data are presented based on the number of individuals (*n* = 354). In cases where discordant phenotypes were present between the bilateral joints of a single patient, the more severe phenotype was used for categorization.

IQR, interquartile range; TMJOA, temporomandibular joint osteoarthritis; DDwR, disc displacement with reduction; DDwoR, disc displacement without reduction.

**Fig. 2:**
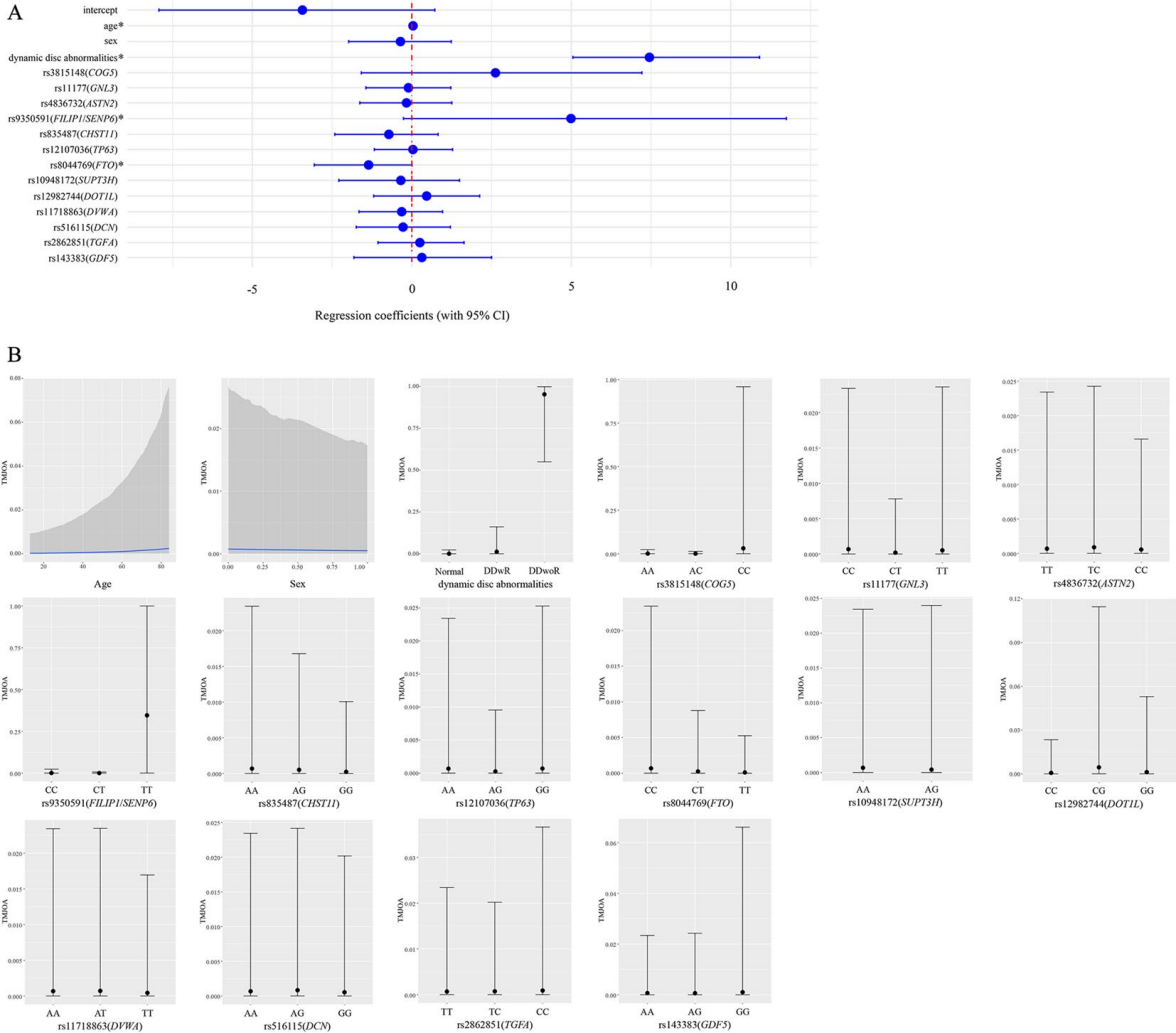
Multivariate analysis based on Bayesian modeling. (**A**) Regression coefficients of the linear component and the intercept. Blue plots and error bars indicate the estimated coefficients and 95% confidence intervals (CI). Regression coefficients with positive and negative values denote the positive and negative impacts on the pathology of TMJOA, respectively. *Covariates with posterior probability greater than 0.95. (**B**) Posterior probabilities of each covariate on the occurrence of TMJOA. Regarding SNPs, allele types were compared. In each graph, the reference allele is represented by the left-most allele. Plots and error bars indicate the estimated coefficients and 95% CI. DDwR, disc displacement with reduction; DDwoR, disc displacement without reduction; TMJOA, temporomandibular joint osteoarthritis.

Multivariate analysis was conducted to investigate the relationship between the presence of TMJOA and the explanatory variables. We constructed a logistic regression model assuming individual-level random effects utilizing Bayesian modeling to consider both individual-related parameters including age, sex, and genomic alleles, and joint-related parameters such as disc displacement. To construct the regression model, pairwise correlation coefficients between the variables were calculated (Supplementary Table 1). High correlations were observed between dynamic disc abnormalities and positional disc abnormalities, and alleles rs3815148 and rs4730250 in *COG5* and *DUS4L*, respectively (Pearson’s *R* = 0.79 and 0.98, respectively); therefore, positional disc abnormalities and rs4730250 were excluded from the model. Furthermore, rs6094710 in *NCOA3* and rs10492367 in *KLHDC5*/*PTHLH* were excluded from the model because no variations were observed in any of the subjects. The explanatory variables in the model included age, sex, presence of dynamic disc abnormalities, and the alleles at the following loci: rs3815148 in *COG5*, rs11177 in *GNL3*, rs4836732 in *ASTN2*, rs9350591 in *FILIP1/SENP6*, rs835487 in *CHST11*, rs12107036 in *TP63*, rs8044769 in *FTO*, rs10948172 in *SUPT3H*, rs12982744 in *DOT1L*, rs11718863 in *DVWA*, rs516115 in *DCN*, rs2862851 in *TGFA*, and rs143383 in *GDF5*. The results of the multivariate analysis indicated that age, dynamic disc abnormalities, SNPs in an intergenic region between *FILIP1* and *SENP6* (rs9350591), and the *FTO* gene (rs8044769) showed non-zero regression coefficients of the linear component with posterior probability above 0.95, which indicates the association with the pathology of TMJOA (Fig. 2, and Supplementary Tables 2 and 3). The expected false discovery rate (eFDR) was low (2.4%), suggesting that only a small proportion of the detected associations are expected to represent false positives despite the simultaneous evaluation of multiple SNPs (Supplementary Table 2). Additionally, sensitivity analyses using additive and dominant/recessive genotype models showed broadly similar patterns to those of the primary genotypic model (Supplementary Tables 2 and 3). For representative loci such as rs8044769 in *FTO* (minor allele frequency = 0.30), the estimated power exceeded 0.80 for odds ratios around 1.7, whereas smaller effects for lower-frequency variants such as rs9350591 between *FILIP1* and *SENP6* (minor allele frequency = 0.15) were likely underpowered (Supplementary Table 4). Based on these statistical findings, the potential functional implications of rs9350591 and rs8044769 were further explored using in silico analyses.

**Fig. 3:**
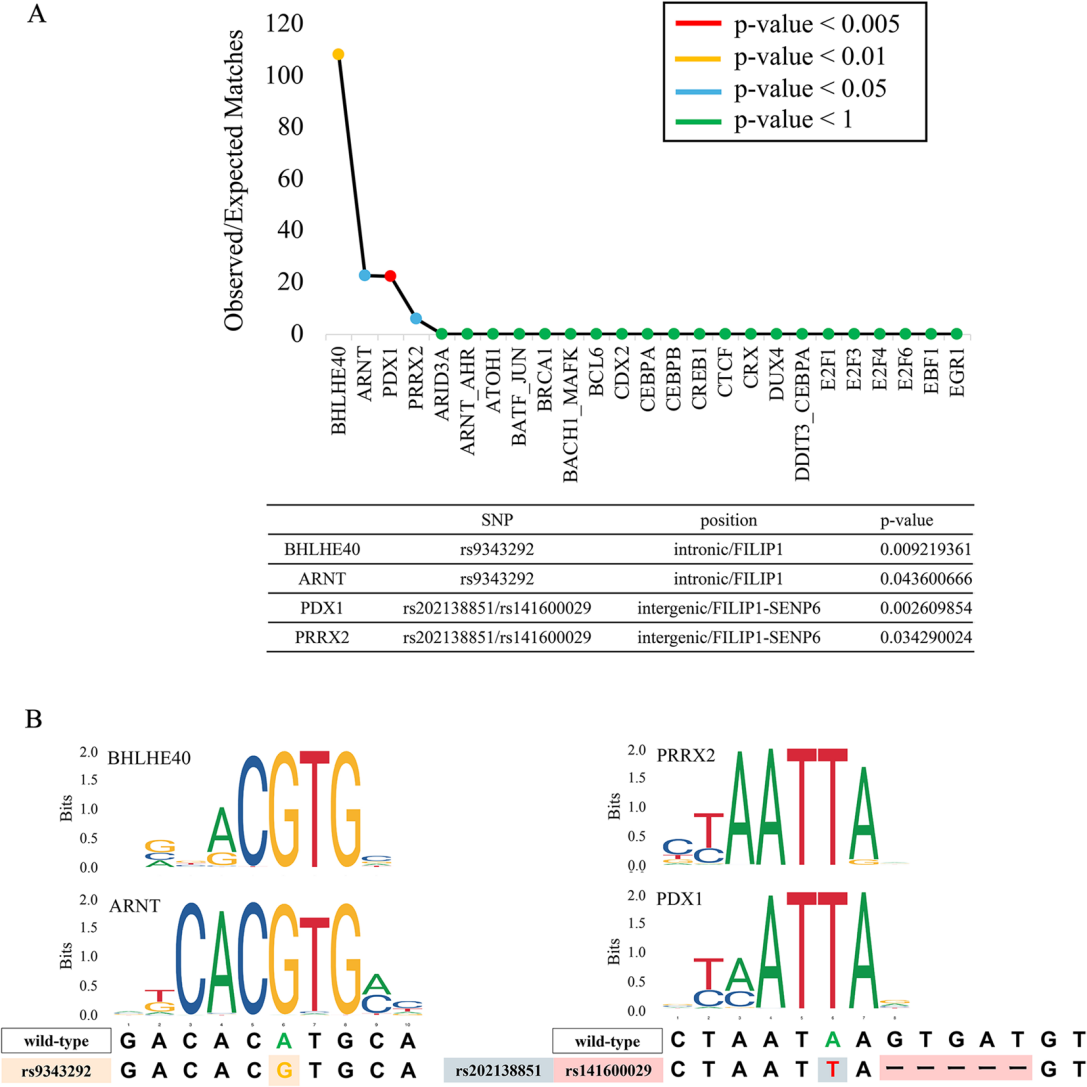
Prediction of transcription factors affected by the polymorphisms. (**A**) TF enrichment analysis of 34 polymorphisms in the region spanning *FILIP1* and *SENP6*. The Y-axis indicates the proportion of observed matches against the expected value for each TF motif. (**B**) Motif-based analysis of the four enriched TFs. The Y-axis shows the trend for each nucleotide. Nucleotides affected by polymorphisms are highlighted in color. TF, transcription factor.

**Fig. 4:**
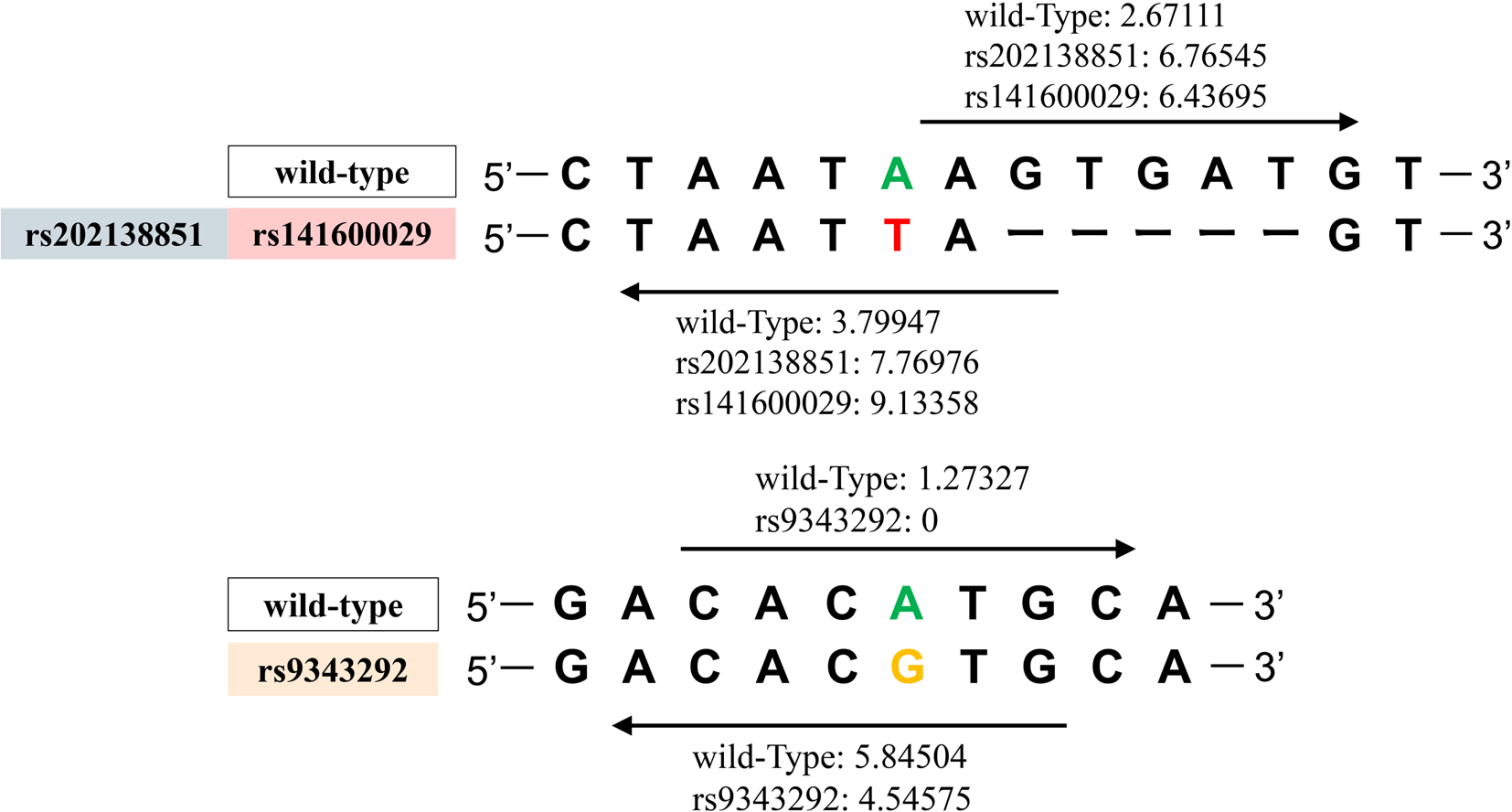
sTRAP analysis. Each score indicates the predicted value of TF-binding motif. Arrows show the predicted TF-binding motifs. TF, transcription factor.

In silico analysis of rs9350591 and rs8044769 revealed that rs9350591 is located in an intergenic region spanning approximately 100,000 base pairs. Assuming linkage disequilibrium in the Japanese population, we investigated other variations that co-occurred with rs9350591, using the jMorp database^30^. Based on the jMorp database, thirty-four candidate polymorphisms in linkage disequilibrium with rs935059 were detected in the region between *FILIP1* and *SENP6* and an intronic region of *FILIP1* (Table 2). In silico screening using RegulomeDB was performed for the thirty-four candidate polymorphisms. Two polymorphisms, rs202138851 and rs141600029, scored the highest probability for regulatory sites (1 and 0.927, respectively) and had the highest evidence category 1b, which indicates corroboration by multiple evidence such as expression-quantitative trait loci (eQTL) data, TF binding, binding motif, DNase footprint, and DNase peak (Table 2). In addition, thirteen polymorphisms, including rs9350591, were rated as category 1f because the eQTL data indicated an association with the expression of flanking genes. To search for TFs binding to the location of the variations, the SNP2TFBS database was employed^32^. Our findings indicate that the TFs ARNT2 and BHLHE40 can bind to the intronic site containing rs9343292 in *FILIP1*. Additionally, the binding of PDX1 and PRRX2 may be influenced by intergenic variants rs202138851 and rs141600029 (Fig. 3). Affinity change prediction with TRAP suggested the possibility that the mutation rs9343292 may moderately attenuate affinity for TFs, while rs202138851 and rs141600029 appear to enhance affinity (Fig. 4).

**Table 2:** Exploration of polymorphisms co-occurring with rs935059 and their evidence as TF-binding sites and eQTL.

Polymorphisms	jMorp			RegulomeDB	
	Distance	Allele frequency	*r* ^2^	Rank	Score
rs9350591	0	0.13	1	1f	0.55436
rs9343292	−38,871	0.13	1	1f	0.66703
rs202138851	3443	0.13	1	1b	1
rs9359127	6118	0.13	1	1f	0.55324
rs141600029	3444	0.13	1	1b	0.92754
rs12207675	−3786	0.13	1	1f	0.55436
rs9359128	8929	0.13	1	7	0.51392
rs144983080	−30,005	0.13	1	7	0.51392
rs13192994	−2265	0.13	1	6	0.14
rs11964634	−21,727	0.13	1	7	0.51392
rs9343299	−1682	0.13	1	7	0.51392
rs9341526	−5138	0.13	1	1f	0.55436
rs12200169	−4406	0.13	1	7	0.51392
rs11963619	11,538	0.13	1	1f	0.55324
rs9352215	−1646	0.13	1	7	0.51392
rs35985089	−39,845	0.13	1	1f	0.66703
rs72877553	−25,869	0.13	0.99	6	0.09
rs35395163	−7526	0.13	0.99	1f	0.55436
rs34222172	−7805	0.13	0.99	5	0.01
rs9359125	−10,223	0.13	0.99	6	0.74073
rs56236638	−25,014	0.13	0.99	7	0.51392
rs12190734	−9543	0.13	0.99	1f	0.22271
rs9343297	−6830	0.13	0.99	1f	0.55436
rs12202443	−2913	0.13	0.99	1f	0.55436
rs28773819	−24,818	0.13	0.99	7	0.51392
rs143730629	12,470	0.13	0.99	6	0.23
rs139176009	−30,122	0.13	0.99	6	0.22339
rs9360913	−17,208	0.13	0.99	7	0.51392
rs10943249	−42,308	0.13	0.99	1f	0.55436
rs35879077	2372	0.13	0.99	5	0.48781
rs35520146	−33,066	0.13	0.98	6	0.74429
rs3056520	−36,578	0.13	0.97	3a	0.66784
rs59226965	4459	0.12	0.91	7	0.18412
rs59493141	−41,174	0.11	0.81	4	0.60906
rs12211255	−53,197	0.14	0.81	1f	0.55436

r^2^ is the correlation coefficient between the rs9350591 polymorphism and the other polymorphisms. Ranks were determined based on the evidence of TF-binding sites. The RegulomeDB model score ranged from 0 to 1, with 1 being the most likely regulatory variant. RegulomeDB ranks^31^ 1b: multiple pieces of evidence such as eQTL data, TF binding, binding motif, DNase footprint, and DNase peak; 1f: eQTL data indicating an association with the expression of flanking genes; 3a: TF binding + any motif + chromatin accessibility peak; 4: TF binding + chromatin accessibility peak; 5: TF binding or chromatin accessibility peak; 6: motif hit; 7: other.

eQTL, expression-quantitative trait loci; TF, transcription factor.

Another TMJOA-associated allele, rs8044769, in *FTO*, was found in a deep intronic region between the first and second exons (NM_001080432(FTO): c.46-4917T > C). According to the jMorp database, there are nine candidate polymorphisms in linkage disequilibrium with rs8044769 (r^2^ > 0.8; Table 3), and these nine polymorphisms are in the intronic regions of *FTO*. ESEfinder predicted a greater decrease of 3’ splice site and branch site scores for rs8047395 than those for rs8044769 (Table 4). Also, SpliceAI predicted a weak gain of 5’ donor activities for rs8047395, while no change was observed in the prediction score for rs8044769 (Table 4). In silico analyses in the present study predicted that the co-occurring mutation rs8047395 may affect mRNA splicing in *FTO*.

**Table 3:** Exploration of polymorphisms co-occurring with rs8044769.

Polymorphisms	Distance	Allele frequency	*r* ^2^
rs8044769	0	0.68	1
rs11075987	−23,974	0.68	0.98
rs61535350	−15,360	0.68	0.97
rs10852521	−34,170	0.68	0.97
rs1861866	−34,795	0.68	0.97
rs8055197	−35,979	0.68	0.97
rs9922047	−32,855	0.68	0.97
rs8047395	−40,612	0.68	0.94
rs11383210	−39,666	0.67	0.91
rs17817288	−31,371	0.65	0.85

r^2^ is the correlation coefficient between rs8044769 and each polymorphism.

**Table 4:** Splicing site prediction using ESEfinder and SpliceAI for rs8044769 and rs8047395.

Sequences of 3’ splicing site (branch site)	ESEfinder		SpliceAI for rs8044769			
	3’ splice site	Branch site	Acceptor loss	Donor loss	Acceptor gain	Donor gain
5’-TGACACGGCTGAAGAGTCAGGAG*TGGGA***C***G-3’*	2.497	0	0	0	0	0
5’-TGACACGGCTGAAGAGTCAGGAG*TGGGA***T***G-3’*	2.3444	0	0	0	0	0

Sequences of 3’ splicing site (branch site)	ESEfinder		SpliceAI for rs8047395			
	3’ splice site	Branch site	Acceptor loss	Donor loss	Acceptor gain	Donor gain
5’-TGGAGTTGAGAGCAT*GTGC***G***AA*CTTGAGAA-3’	3.1938	0.4687	0	0	0	0
5’-TGGAGTTGAGAGCAT*GTGC***A***AA*CTTGAGAA-3’	0	0.0588	0	0	0	0.01

Sequences predicted by ESEfinder as putative 3’ splicing sites are shown and the predicted branch site are underlined. Bold letters indicate nucleotides affected by these polymorphisms.

## Discussion

In recent years, there have been many studies on genome analysis, and the identification of polymorphisms in TMJOA will facilitate the identification of suitable signaling pathways for therapeutic intervention and preoperative risk diagnosis. This will allow the development of tailored drugs that match the genetic predisposition of patients, thereby improving the efficacy of treatment and reducing the likelihood of adverse outcomes.

SNPs are polymorphisms that occur due to single-nucleotide substitutions caused by point mutations and are currently used as markers in many genomic studies. Although the involvement of genetic factors in the onset of OA has been reported, most studies have focused on the knee and hip joints, and there have been only a few genomic studies on the TMJ. One reason for this is the excessive cost of MR imaging examinations. Few clinical studies have been conducted after the diagnosis of TMJOA using MR imaging, which is considered the gold standard for diagnosing TMJ disorders. Furthermore, the difficulty in collecting data on normal TMJs using MR imaging is also thought to be a factor hindering the development of this research. In the present study, genomic data were collected from 354 patients who underwent MR imaging; more than half of these patients did not have TMJOA. Multivariate analysis was conducted by adjusting for the effects of disc abnormalities and other clinical information. The identified TMJOA-associated SNPs, including rs9350591 (intergenic region between *FILIP1* and *SENP6*), and rs8044769 (*FTO*), should therefore be interpreted as potential genetic risk factors for TMJOA among patients with TMD. Therefore, the functions of SNPs rs9350591 and rs8044769 were predicted using in silico analyses. On the other hand, the univariate analysis showed statistically significant differences in rs12107036 in *TP63* (Table 1), but rs12107036 was not detected in the multivariate analysis (Fig. 2A). This may be because the effect of rs12107036 was removed by adjustment for other variants.

Rs9350591 and rs8044769 are in the intergenic region between *FILIP1* and *SENP6* and the intronic region of *FTO*, respectively. Rs9350591 has been previously identified as a variant associated with hip OA^37^. However, another study found no correlation between the rs9350591 genotype and gene expression in OA and non-OA hip cartilages obtained through total joint replacement, although the expression of *SENP6* and *MYO6* in OA cartilage was significantly lower than that in non-OA cartilage^38^. The authors posited that the collected hip cartilage samples reflected end-stage OA and suggested that rs9350591 might be related to chondrogenesis at an earlier stage of OA. In the present study, we demonstrated that the TFs BHLHE40 and ARNT2 could bind to the site where rs9343292, co-occurring with rs9350591, is located. BHLHE40, also known as differentiated embryo chondrocyte expressed gene-1 (DEC1), has been previously suggested to promote chondrogenic differentiation and engages in dysregulated gene expression in OA cartilage^39–41^. ARNT2 is also known as hypoxia-induced factors 2β (HIF-2β), which forms a heterodimer with HIF-2α^42^. HIF-2α expression is higher in osteoarthritic cartilages than in healthy cartilage of mice and humans, and HIF-2α functions as a catabolic factor in the osteoarthritic process. Our affinity change prediction suggested the possibility that the rs9343292 mutation may moderately attenuate affinity for TFs. Taken together, it is possible that co-occurring variants of the TMJOA-associated SNP rs9350591, rather than rs9350591 itself, may influence TF binding to the chromosome.

Takaoka et al. previously reported an association between rs8044769 in *FTO* and TMJOA using univariate analysis^26^. In the present study, we applied multivariate modeling and performed in silico predictions regarding linkage disequilibrium. Our results confirmed that rs8044769 remained associated with TMJOA after adjustment for clinical covariates. Furthermore, rs8047395, which is in linkage disequilibrium with rs8044769, was predicted to be a more plausible candidate, possibly contributing to the pathology, rather than rs8044769 itself. FTO is an RNA demethylase that regulates fat mass, adipogenesis, and energy homeostasis. It is ubiquitously found in the human body and is expressed in joint-related tissues such as cartilage, tendons, ligaments, meniscus, and synovium^43^. It has been reported that conditional knockout of *FTO* in cartilage cells increases methylated mRNA of *SMAD2*, which reduces the expression of the extracellular matrix and leads to the progression of OA^44^. The results of the present study demonstrate that deep intronic variations in *FTO* are associated with TMJOA and may influence its binding affinity with splicing-related molecules. To generate mature mRNAs, precise splicing is necessary, despite the presence of intronic pseudo exons. However, some intronic mutations can perturb the machinery. For example, an intronic mutation, IVS4 + 866 C > T, in the *IKBKG* locus was shown to create a new splicing donor site and pseudo exon, which caused a frameshift mutation^45^. In recent decades, some relatively long introns have been found to be removed by recursive splicing^46,47^. Kelly et al. concluded that transcripts from > 60% of active genes undergo recursive splicing or stepwise removal of intronic sequences^47^. However, further experiments are required to determine whether intronic variations in *FTO* generate pseudo exons or affect splicing efficacy, resulting in downregulation of the mature FTO protein.

This study has some limitations. First, factors other than genetics cannot be ruled out in the pathogenesis of TMJOA. TMJOA, which develops in adolescence, may be more strongly influenced by genetic factors than TMJOA in adulthood, which is likely to be synergistically affected by multiple factors. In addition, we could not evaluate the effects of mastication-related parameters such as speed, duration, cycle of mastication, dentition deficiency, and denture use, because they were not included as explanatory variables in the models used in this study. The second limitation of this study was that patients without TMJOA were not asymptomatic or healthy; rather, it comprised patients without any degenerative bone changes in the condylar head who complained of the symptoms. Third, a limited number of SNPs were investigated. In OA of the limb joints such as the knee and hip, there have been reports of associations with more SNPs than those investigated in this study. However, because of the limitations on the number and combinations of SNPs that can be investigated simultaneously with microarray-based genotyping, only 16 SNPs were analyzed in this study. Further large-scale studies are required to confirm these findings. Finally, this study lacks an independent validation cohort and is limited to a Japanese population. To address potential population stratification, all participants were recruited from a single center and were of shared Japanese ancestry. While this homogeneity reduces the likelihood of significant ancestral confounding between cases and controls, it does not entirely eliminate it. Furthermore, the generalizability of our findings to other ethnic groups remains to be established.

## Conclusions

We found that the SNPs rs9350591 and rs8044769 in the intergenic region between *FILIP1* and *SENP6* and in the *FTO* gene might be associated with TMJOA through co-occurring polymorphisms.

## Supplementary Information

Below is the link to the electronic supplementary material.

Supplementary Material 1

Supplementary Material 2

Supplementary Material 3

Supplementary Material 4

## Data Availability

The datasets generated and/or analyzed during the current study are available from the corresponding author on reasonable request.
